# Machine learning analysis reveals tumor stiffness and hypoperfusion as biomarkers predictive of cancer treatment efficacy

**DOI:** 10.1016/j.tranon.2024.101944

**Published:** 2024-03-28

**Authors:** Demetris Englezos, Chrysovalantis Voutouri, Triantafyllos Stylianopoulos

**Affiliations:** Cancer Biophysics Laboratory, Department of Mechanical and Manufacturing Engineering, University of Cyprus, Nicosia, Cyprus

**Keywords:** Ultrasound imaging, Shear wave elastography, Chemo-immunotherapy, Precision oncology, Biomarkers

## Abstract

In the pursuit of advancing cancer therapy, this study explores the predictive power of machine learning in analyzing tumor characteristics, specifically focusing on the effects of tumor stiffness and perfusion (i.e., blood flow) on treatment efficacy. Recent advancements in oncology have highlighted the significance of these physiological properties of the tumor microenvironment in determining treatment outcomes. We delve into the relationship between these tumor attributes and the effectiveness of cancer therapies in preclinical tumor models. Utilizing robust statistical methods and machine learning algorithms, our research analyzes data from 1365 cases of various cancer types, assessing how tumor stiffness and perfusion influence the efficacy of treatment protocols. We also investigate the synergistic potential of combining drugs that modulate tumor stiffness and perfusion with standard cytotoxic treatments. By incorporating these predictors into treatment planning, our study aims to enhance the precision of cancer therapy, tailoring treatment to individual tumor profiles. Our findings demonstrate a significant correlation between stiffness/perfusion and treatment efficacy, highlighting a new way for personalized cancer treatment strategies.

## Highlights

• Identified tumor stiffness and hypoperfusion as predictive biomarkers for treatment efficacy.
• Utilized advanced machine learning (XGBoost) to analyze data from 1365 tumor cases.
• Demonstrated the effectiveness of mechanotherapeutic drugs in enhancing treatment outcomes.
• Employed Shear Wave Elastography and Contrast-Enhanced Ultrasound for non-invasive tumor assessment.
• Revealed potential for personalized cancer therapy by integrating biomechanical biomarkers.

## Graphical abstract

Biophysical properties of solid tumors, including tumor stiffness measured as elastic modulus values with ultrasound shear wave elastography (SWE) and perfusion, quantified with contrast-enhanced ultrasound (CEUS), can predict tumor response to chemo-immunotherapy with the use of advanced statistical and machine learning models.

**Figure:**
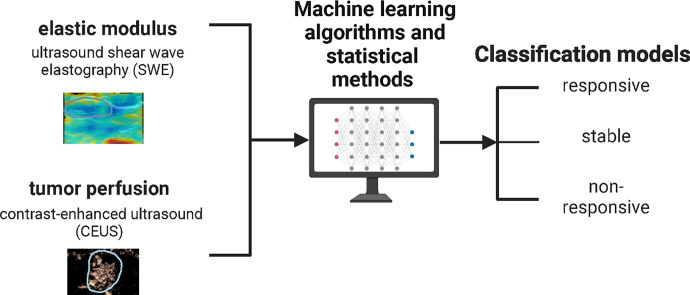

## Introduction

Pathophysiological properties of the tumor microenvironment (TME) can pose major barriers to the efficacy of cancer therapies [1]. Among these properties, mechanical aspects of the TME, and in particular the abnormal elevation of tumor stiffness and the lack of functional tumor blood vessels to carry blood, known as hypoperfusion (or ischemia), have shown not only to negatively affect cancer therapy but also serve as valuable indicators for predicting treatment outcomes [2], [3], [4], [5], [6], [7]. Tumors are understood to be heterogeneous in both perfusion and stiffness, not just across cancer types, but even within a single type and within a single tumor itself during progression. In fibrotic tumors, also known as desmoplastic tumors, impaired perfusion is primarily caused by vessel compression, which reduces the effective cross-sectional area of the vessels, resulting in hypoperfusion [8], [9], [10], [11]. Tumors with abundant compressed vessels are commonly found in subsets of breast and pancreatic cancers, as well as certain sarcoma subtypes.

Tumors as they grow at the expense of the host tissue exert mechanical forces. Reciprocal forces are also exerted from the host tissue on the tumor. These forces accumulate within the structural components of the tumor [8]. These forces are known as solid stress. Tumor stiffness and solid stress in the TME compress intratumoral blood vessels, inducing hypoperfusion. Hypoperfusion leads to hypoxia and poor delivery of medicines, which in turn reduces the effectiveness of cancer therapies. Low blood flow limits not only drug delivery but also the amount of immune cells reaching the tumor, while hypoxia makes the TME immunosuppressive and weakens the ability of immune cells to kill cancer cells [12], [13], [14], [15]. Additionally, hypoxia affects the function of T lymphocytes and other immune cells, while low blood flow and the dense/stiff TME create a physical barrier to T cell infiltration into the tumor. Studies have shown that increasing blood flow can reverse these pro-tumor effects and improve the effectiveness of immune checkpoint inhibition [[4], [5], [6],[16], [17], [18], [19], [20], [21], [22], [23], [24], [25]]. Therefore, tumor stiffness and the degree of perfusion have been considered as predictive biomarkers of response to therapy not only in preclinical but also in clinical studies [[5], [6], [7],^9^,[26], [27], [28]]. Predicting patients’ responses to therapy is crucial as therapy can lead to optimized, patient-specific treatments.

Mechanotherapeutic drugs, such as the tranilast, bosentan and ketotifen, employed in our study, work by targeting the TME to modulate its mechanical properties, particularly tumor stiffness. These drugs exert their effects through various mechanisms, including the inhibition of collagen and hyaluronan synthesis and the reduction of fibroblast activation [5,[20], [21], [22], [23]]. By altering the ECM composition, these agents decrease the mechanical resistance within the tumor, thus reducing its stiffness. This reduction in stiffness not only facilitates better penetration and distribution of cytotoxic agents within the tumor mass but also improve the efficacy of immunotherapeutic approaches by enhancing immune cell infiltration into the tumor.

In this study, we explored the significance of tumor stiffness and hypoperfusion as predictive biomarkers in cancer treatment. Utilizing murine tumors from various cancer cell lines, including breast cancer (4T1 and E0771), fibrosarcoma (MCA205), osteosarcoma (K7M2), and melanoma (B16F10), we investigated how these physical properties of tumors influence the efficacy of treatment protocols, including chemotherapy, immunotherapy, or their combination. Our research aimed to uncover the potential of stiffness and hypoperfusion as indicators of a tumor's response to therapy, proposing that predictive biomarkers based on these two properties warrant further development. [5,[20], [21], [22], [23]] In our analysis, we utilized the XGBoost [29] algorithm, a leading method within the realm of supervised machine learning. XGBoost is particularly favored for its efficiency, scalability, and performance in predictive accuracy across a wide range of data types and domains [30], [31], [32]. It operates by constructing an ensemble of decision trees in a gradient boosting framework, which iteratively minimizes errors. This technique is adept at handling complex datasets with noise and missing values, making it ideal for our analysis involving diverse and high-dimensional data from cancer research.

In addition, we employed two advanced ultrasound imaging techniques, namely shear wave elastography (SWE) and contrast-enhanced ultrasound (CEUS), to measure the elastic modulus and perfusion of tumors, respectively. SWE is a non-invasive ultrasound technology that measures the stiffness of tissues in real time. It does so by generating shear waves within the tissue and then calculating the speed of these waves. The speed is directly related to tissue stiffness, with faster speeds indicating stiffer tissues. This technique is particularly useful for assessing the mechanical properties of tumors, providing insights into tumor stiffness without the need for invasive procedures. CEUS involves the intravenous administration of microbubbles as ultrasound contrast agents, which are microbubbles that enhance that are imaged by the ultrasound signalsystem. This technique allows for real-time imaging of blood flow and tissue perfusion within the tumor. By observing the distribution and intensity of the contrast agent, CEUS can quantify the extent of blood flow and identify areas of hypoperfusion or ischemia within the tumor.

## Materials and methods

### Dataset

**Fig. 1:**
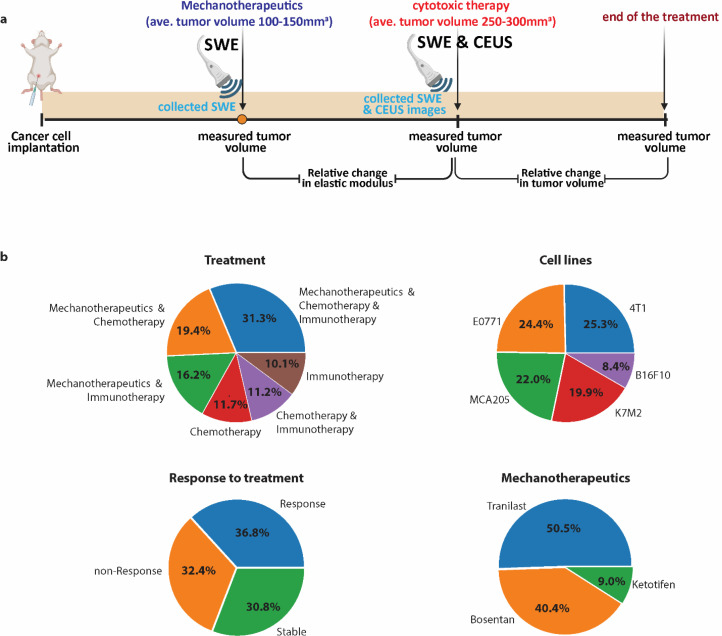
Dataset: a Schematic of the Experimental Protocol and Imaging Techniques. The figure outlines the experimental setup, including the treatment regimen and the timing for employing SWE and CEUS to assess the mechanical and vascular characteristics of tumors. Initially, tumors were allowed to reach a volume of approximately 100–150 mm³ before the administration of a mechanotherapeutic agent (tranilast, bosentan or ketotifen). Once tumors reached a volume of 250–300 mm³, cytotoxic treatments, consisting of chemotherapy, immunotherapy or their combination, were initiated. SWE was utilized to evaluate the elastic modulus of tumors. CEUS was subsequently applied to monitor the perfusion status and detect areas of hypoperfusion within the tumor. b Composition of collected dataset showing the percentage of the treatment procedures, cell lines, responsive tumors, and the optional administration of medication for mechanotherapy treatment prior to cytotoxic therapy.

A dataset of 1365 murine tumors was collected from our past experiments [5,[20], [21], [22], [23]]. The data were produced from several tumor types, including breast cancer (4T1 and E0771), fibrosarcoma (MCA205), osteosarcoma (K7M2), and melanoma (B16F10) treated with chemo-immunotherapy. It is important to note that the implantation of these diverse cell lines generally yielded comparable effects in terms of tumor progression, thereby establishing a common benchmark for analysis. Out of these cases, prior to cytotoxic therapy, 774 tumors were treated with a mechanotherapeutic agent at their corresponding optimal dose: tranilast 200 mg/kg (Rizaben, Kissei Pharmaceutical, Japan), bosentan 1 mg/kg (Selleckchem, USA) or ketotifen 10 mg/kg (Sigma-Aldrich, USA). Cytotoxic therapy included chemotherapy (Doxil 3 mg/kg or doxorubicin 5 mg/kg), immunotherapy (monoclonal anti-PD-L1 10 mg/kg, clone 10F.9G2 BioXCell, or a cocktail of monoclonal PD-1 10 mg/kg, clone RMP1–14 BioXCell and monoclonal CTLA-4 5 mg/kg clone 9D9, BioXCell) or the combination of chemo- and immunotherapy. The other 383 tumors were treated only with cytotoxic therapy. The treatment protocol is shown in Fig. 1a.

At specific times during tumor progression the elastic modulus and perfusion of the tumors were measured with non-invasive ultrasound system SWE and CEUS, respectively. The average values of the elastic modules from the entire region of the tumor and from two different planes were taken to quantify the overall elastic modulus of a tumor. Tumor perfusion was quantified as the area of the tumor that contrast agent were detected over the total area of the tumor, which we refer to as the normalized perfused area. Mechanotherapeutic treatment (tranilast, bosentan or ketotifen) was initiated daily treatment when tumors reached a size of ∼100–150 mm [3] for the entire period of the experimental protocol and the administration of the cytotoxic treatment regimen, consisting of chemotherapy, immunotherapy or their combination, started when tumors reached a volume of 250–300 mm^3^ (Fig. 1a). Tumor volume was measured with ultrasound system B-mode images every second day. Responsive, stable and non-responsive tumors were defined by their relative tumor volume change, employing the RECIST (Response Evaluation Criteria in Solid Tumors) criterion [33], occurred between the time of the cytotoxic therapy administration and the end of the treatment (Fig. 1a): Responsive (relative tumor volume change <1.2), stable (1.2 <relative tumor volume change <2), and non-responsive (relative tumor volume change >2).

Data were split into training and testing sets. The training set was used to train all models developed and the testing set was used to evaluate the performance of the best performing models from the previous step on unseen data. The training and testing set consisted of 80 % and 20 % of the data, respectively. Moreover, to maintain data integrity and avoid any potential bias, we ensured that the data used during the machine learning training and testing phases were balanced representations of the cell lines; that is, by performing random data shuffling, we empirically assessed that each cell line has approximately equal representation within both the training and testing datasets. This means that every data point is randomly rearranged, breaking any initial order that might introduce bias or affect the machine learning model's learning process. The goal is to ensure that when the dataset is split into training and testing sets, each set is a balanced representation of the overall data, preventing model overfitting to specific patterns that might only exist due to the initial data ordering.

To maintain data integrity and avoid any potential bias during the machine learning training and testing phases, we took meticulous steps to ensure that the data used were balanced representations of the cell lines involved in our study. This balance is crucial for several reasons. A balanced dataset helps prevent the machine learning model from developing a bias toward one or more cell lines, which could skew the results and reduce the generalizability of the model. Balanced data ensures that the model is equally exposed to the variety of patterns and characteristics across different cell lines. This enhances the model's ability to learn and accurately predict outcomes across a broad spectrum of tumor types. By ensuring that each cell line has approximately equal representation within both the training and testing datasets, we enhance the robustness of our model. This means our model is better equipped to handle new, unseen data while maintaining its predictive accuracy. To achieve this balance, we employed random data shuffling, a technique that randomly distributes data points across the training and testing sets. This method ensures that each cell line is represented proportionately, thus preserving the integrity of our dataset and the validity of our findings.

### Ultrasound imaging

The SWE images were captured using a Philips EPIQ Elite Ultrasound system equipped with a linear array transducer (eL18–4). This transducer measures the velocity of 2D shear waves as they travel through tissue perpendicular to the original acoustic impulse. The result is a color-mapped elastogram (in kPa) overlaid on a B-mode anatomical ultrasound image. The color spectrum, ranging from blue (soft tissue) to red (hard tissue), provides information about tissue stiffness. Representative images are shown in the Supplementary Figure 1A. A confidence map is also used to verify the quality of the shear waves within a user-defined region of interest (ROI). The average value of stiffness in the tumor region is automatically generated by the system using default scanner settings and expressed in kPa. SWE was performed at two different planes of the tumor, one along the smallest diameter and the other along the largest diameter. The average value of both planes was used for our analysis.

The use of ROI in ultrasound imaging, particularly in SWE, serves a vital role in validating the quality of shear waves generated and captured within the tissue. This process involves selecting a specific area on the ultrasound image, where the shear waves' propagation is expected to be the most indicative of the tissue's mechanical properties. By focusing on this area, can ensure that the measurements obtained reflect the true stiffness of the tissue under investigation. Verification within the ROI involves several steps. A predefined area is selected on the ultrasound image where the shear waves are expected to be uniform and indicative of the tissue's stiffness. The ultrasound system then assesses the quality of the shear wave signals within the ROI. This includes checking for uniform propagation of the waves, consistent wave velocity, and absence of artifacts that could affect the accuracy of stiffness measurements. Many advanced ultrasound systems, including ours, provide a confidence map, which visually represents the quality of the shear wave data. High confidence areas indicate reliable data, whereas low confidence areas might suggest the presence of artifacts or inconsistencies in wave propagation. Only data from high-confidence areas within the ROI are used for calculating the tissue's stiffness. This approach minimizes the impact of potential errors or artifacts, ensuring that the elastography results are both accurate and reliable.

The same ultrasound system was used for CEUS to evaluate tumor blood flow and perfusion parameters. This was done after injecting an 8 μl bolus of Sonovue (Lumason in the USA) contrast agent (Bracco, Geneva, Switzerland). Sonovue consists of hexafluoride microbubbles enclosed in a phospholipid shell, with an average diameter of 2.5 μm. The contrast agent was injected retro-orbitally, as tail vein injections of microbubbles tend to have high variability. The choice of retro-orbital injection over tail vein injection in our study aims to reduce variability and enhance consistency in delivering agents into the systemic circulation of rodents. Retro-orbital injection is preferred for its reliability and lower stress on animals, ensuring uniform dosing and better experimental outcomes. This method supports our commitment to maintaining data integrity and reproducibility in our research. Before each ultrasound scan, the mice were anesthetized with an intraperitoneal injection of Avertin (200 mg/kg). The ultrasound scanning of tumors was conducted using the linear array transducer L12–5. The image analysis was performed offline using custom software, representative images shown in the Supplementary Figure 1B For our analysis, we calculated the normalized perfused area at the time when the image intensity reaches its peak. This was done by dividing the number of pixels with microbubble signals by the total number of pixels within the tumor ROI.

In our machine learning model development, while we generally adhere to the principle of excluding features that exhibit high correlation to mitigate multicollinearity issues, we made a deliberate exception for certain features such as elastic modulus and normalized perfused area. This decision is grounded in the recognition that, although these features are intrinsically linked, each provides unique and complementary information critical for predicting treatment outcomes in our context. We relied on preliminary analysis and existing literature that suggests the combined use of these parameters can notably enhance predictive models' accuracy in oncological studies. For instance, studies have shown that the mechanical properties of tumors, as measured by elastic modulus, and their vascular characteristics, as indicated by perfused area, contribute to the tumor's response to therapies.

## Statistical analysis

### Feature importance

To predict tumor response to treatment, it is important to apply feature engineering to learn which of the characteristics that describe the tumors are important and will statistically contribute to the prediction. One way to identify the features that contribute the most is to perform post-analysis in predicting outcomes of a logistic regression model that classifies the tumors into responsive, stable and non-responsive. This analysis involves the examination of the coefficients associated with each feature used in the model, as well as interpreting their corresponding odds ratios. By doing so, we can determine which features made a statistically significant contribution and whether it was in a positive or negative direction. A post-analysis tool (e.g., Shap [34]) can be used as well to explain the output of a machine learning model to understand the contribution of each feature.

The post-analysis tool, specifically SHAP (SHapley Additive exPlanations), is a method for explaining the output of machine learning models. SHAP values provide insights into how each feature in our dataset contributes to the model's predictions, offering a way to interpret complex model behaviors. By utilizing SHAP, we can understand the impact of each feature on the model's decision-making process, allowing for a more transparent and interpretable analysis of our machine learning outcomes [35], [36], [37].

Additionally, it is common in feature engineering to exclude a feature if it shows a high correlation with another. Although this approach is beyond the scope of our current study, it is relevant to note that features such as elastic modulus and normalized perfused area are intrinsically linked to each other [5,23]. Despite this inherent correlation, our findings in classification models underscore a significant observation: the inclusion of additional tumor-related information, even when it shares a correlation with already present features, can notably enhance the model's accuracy. This suggests that in the context of tumor characteristic analysis, the comprehensive integration of data, regardless of inter-feature correlations, may be beneficial for improving predictive precision.

In our machine learning model development, while we generally adhere to the principle of excluding features that exhibit high correlation to mitigate multicollinearity issues, we made a deliberate exception for certain features such as elastic modulus and normalized perfused area. This decision is grounded in the recognition that, although these features are intrinsically linked, each provides unique and complementary information critical for predicting treatment outcomes in our context. We relied on preliminary analysis and existing literature that suggests the combined use of these parameters can notably enhance predictive models' accuracy in oncological studies. For instance, studies have shown that the mechanical properties of tumors, as measured by elastic modulus, and their vascular characteristics, as indicated by perfused area, contribute to the tumor's response to therapies.

### Statistical comparison among treatment groups

**Fig. 2:**
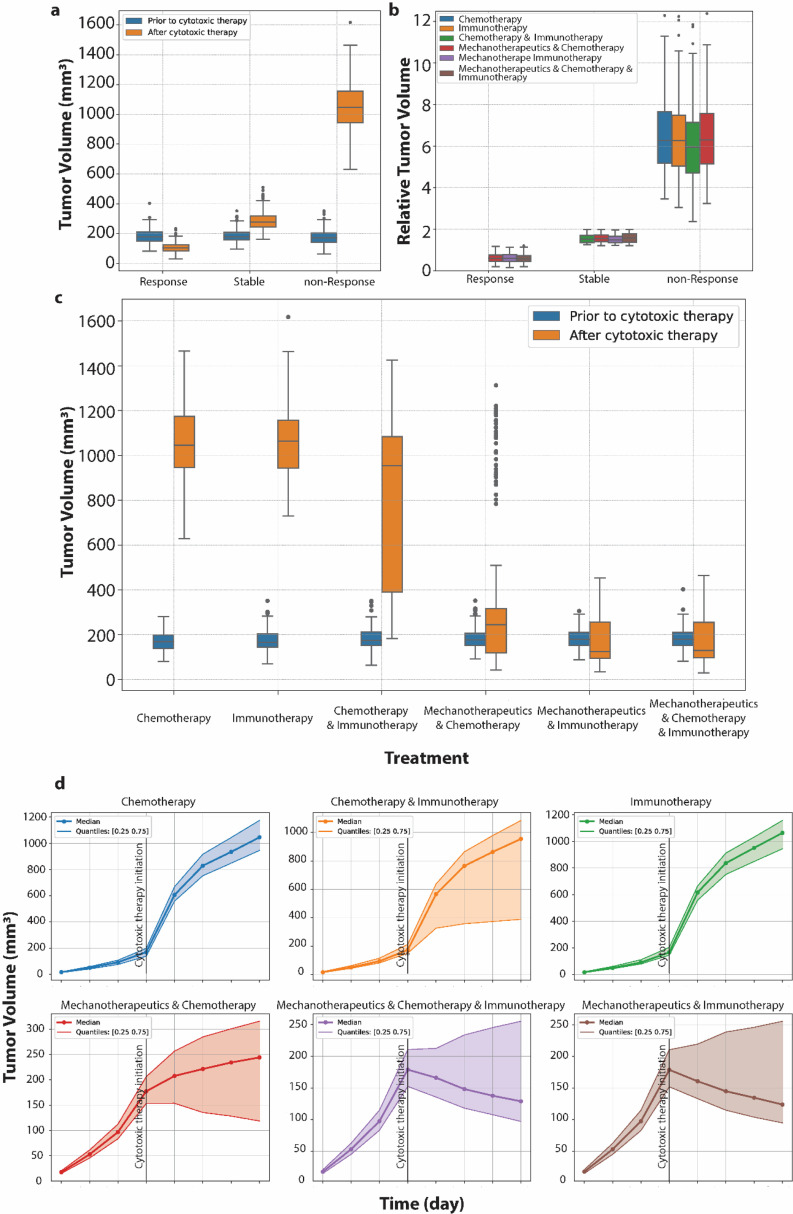
Tumor volume and relative change in tumor volume shorted with respect to response to therapy (a, b) and treatment (c), using a cytotoxic drug (chemotherapy, immunotherapy, or the combination of chemo- and immunotherapy) on cancer cell lines (4T1, E0771, MCA205, K7M2 and B16F10), noting that data for some treatments are not depicted across all response categories because these categories don't have data from these therapies. (d) Tumor volume based on the treatment they received. Median, 0.25 and 0.75 quartiles are presented.

The current analysis, in alignment with previous publications [5,23] demonstrates that the use of mechanotherapeutics prior to cytotoxic therapy leads to reduced tumor stiffness and increased perfusion. This effect subsequently results in better drug delivery within the tumor and improved therapy [5,[20], [21], [22], [23]]. Indeed, tumors treated with mechanotherapeutics and cytotoxic therapy have smaller volumes at the end of the treatment protocol (Fig. 2). Furthermore, we conducted a statistical comparison among the three treatment groups that include mechanotherapeutics followed by chemotherapy, immunotherapy, or their combination, using an ANOVA test [38]. Additionally, during the mechanotherapy phase of the treatment, we administered either tranilast, bosentan or ketotifen to reduce tumor stiffness [5,[20], [21], [22], [23]]. The impact of each of these on each tumor type was also examined through statistical analysis involving ANOVA and Student's *t*-tests [39]. Student's *t*-tests were used for comparing paired groups while ANOVA test was used to compare more than two groups. In cases where three groups are statistically compared with ANOVA test, Tukey's Honest Significant Difference (HSD) was used to assess the difference between individual pairs of group means.

To further elucidate the outcomes of the treatment comparisons, we incorporated a chart detailing the percentage of tumors to increased or decreased elastic modulus for the different treatment groups over the study period (Supplementary Figure 2).

## Classification models

We employed a XGBoost classification model to categorize tumors into one of the three groups: responsive, stable, or non-responsive. To optimize model performance, we conducted cross validation to find the best hyperparameters with different input features: elastic modulus, perfused area, tumor volume, relative tumor volume, therapy and tumor type. Building upon the prior analysis for finding the important features, we can assess and compare the prognostic performance using different feature sets to characterize the tumors.

### Tumor growth prediction

For patient-specific therapies, it is of primary importance to predict the tumor's response prior to the initiation of cytotoxic therapy. To facilitate this, a tumor growth regression model was developed using XGBoost regression that takes as inputs the elastic modulus and normalized perfused area prior to the initiation of the cytotoxic therapy, the treatment protocol that was followed and tumor volume measurements at four distinct time points: 4, 7 and 10 days post implantation of cancer cells and at the day of the initiation of the cytotoxic treatment. With this information, the model predicts how the tumor volume will evolve in the subsequent days, thereby enabling more personalized and effective treatment strategies.

### Uncertainty quantification of tumor growth prediction

To quantify the uncertainty of the tumor growth regression model, a bootstrapping method was implemented. Specifically, we created 100 subsamples from the original training set, each of these with 900 observations drawn with replacement. Consequently, we created 100 distinct XGBoost regressor models that forecast the tumor volumes of each observation in the test set. Out of these 100 predictions, we computed the mean value of each observation and established 95 % confidence intervals. As expected, when predicting future volumes for longer times, we observed an expansion in the width of the confidence intervals.

## Results

### Data analysis

The data were clustered into three classes based on their elastic modulus and normalized perfused area. Supplementary Figure 3 demonstrates that this data separation holds true for all tumor models examined in this study, allowing for their collective analysis. The analysis indicates that these two tumor characteristics explain most of the variability of the data and can be jointly used as biomarkers for predicting whether tumors will respond, stay stable, or not respond to cytotoxic therapy.

This can be validated through the analysis of feature importance (Supplementary Table 1 and Supplementary Table 2). The “response” class serves as the baseline, providing coefficients and p-values to transition from the “response” class to either the “stable” or “non-response” class. For the treatment procedure group, we set the mechanotherapeutics & chemotherapy group as the baseline since it is presented in all three classes. For the interpretation of the results, we employed a 95 % confidence level (significance level *a* = 0.05) to determine feature significance. To transition from the "response" to the "stable" class, the statistically important features are the elastic modulus and normalized perfused area, along with the mechanotherapeutics & Chemotherapy & Immunotherapy. Notably, an increase in elastic modulus contributes to classifying a tumor as "stable" rather than "responsive" (as indicated by a positive coefficient), while an increase in normalized perfused area is associated with a shift towards the "response" class (as suggested by a negative coefficient). Specifically, if all other variables remain constant, the odds of a tumor being classified as "stable" rather than "response" increase by a factor of 1.75 for every 1 kPa increase in elastic modulus. Similar interpretations apply for classifying a tumor as "non-response" rather than "response". However, it is worth noting that the coefficients for elastic modulus and normalized perfused area have larger absolute values in this context, indicating that an increase in elastic modulus or a decrease in normalized perfused area contributes more significantly to a transition from the “response” to the “non-response” class.

To quantify tumor perfusion from CEUS imaging, we calculated the normalized perfused area at the moment when the image intensity reached its peak. This approach is grounded in the principle that peak intensity accurately represents the maximum concentration of contrast agents within the tumor vasculature, providing a snapshot of the highest level of blood flow and perfusion. By assessing perfusion at this peak, we ensure that the evaluation reflects the optimal enhancement of the microvascular network within the tumor, capturing the most significant perfusion status achievable post-contrast injection. This methodology is crucial for distinguishing between areas of adequate perfusion and regions of hypoperfusion within the tumor. Furthermore, the time of peak intensity offers a standardized benchmark for comparison across different scans and subjects, thereby enhancing the reproducibility and reliability of our perfusion measurements

In our analysis of the relationship between tumor stiffness, as quantified by the elastic modulus, and the efficacy of cancer treatments, we identified a statistically significant correlation. Specifically, for every 1 kPa increase in elastic modulus, we observed a 1.75-fold increase in the odds of a tumor responding less favorably to treatment. This factor of 1.75 was derived from logistic regression analysis, a statistical method used to model the probability of a certain class or event existing, such as treatment success or failure, based on one or more predictor variables. This finding underscores the importance of considering tumor stiffness in the development and optimization of cancer treatments. It provides a quantitative basis for the observed clinical challenges in treating highly stiff tumors and reinforces the potential of targeting tumor stiffness as a therapeutic strategy to improve treatment efficacy.

Supplementary Figure 4 provides, through Shap analysis, a post-hoc interpretability analysis. On the y-axis, features are ranked from the most statistically important to the least important. As expected, the elastic modulus is the most important feature, followed by the normalized perfused area. Treatment procedures are following in statistical importance. Additionally, this analysis provides insights into which predictions each feature predominantly influences. For instance, Mechanotherapeutics & Immunotherapy appear to be most influential in predicting responsive tumors. These findings hold true for all tumor types, as the differences between tumor types do not carry significant importance.

### Tumor characteristics

**Fig. 3:**
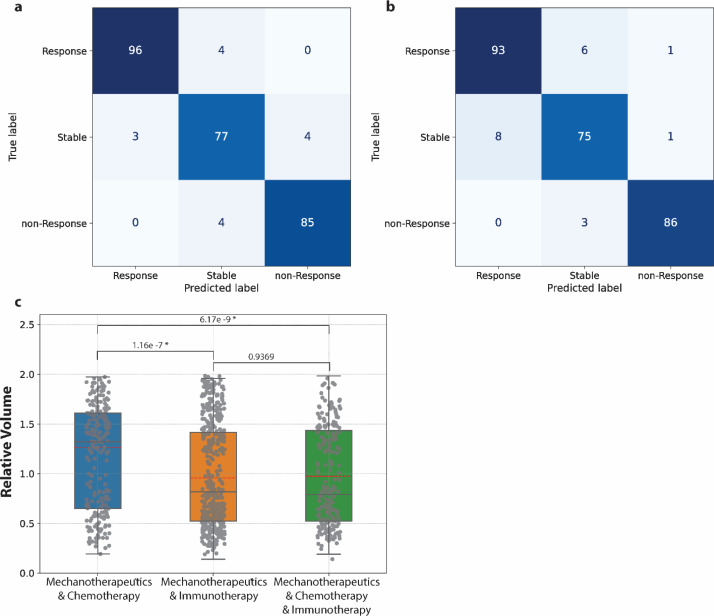
Confusion matrices of the classification models having as input features a) the elastic modulus and normalized perfused area and b) the elastic modulus and the treatment procedure information. c) Statistical comparison between the treatment groups’ relative volume means that include treatment with mechanotherapeutics prior to the initiation of cytotoxic therapy. Means are shown with red dashed lines. P-values of the ANOVA test found to be 5.8110^−10^ which indicates that there are significant differences between the three groups.

Various classification models were developed using XGBoost, employing different sets of input features. The accuracy scores of these six models are presented in Table 1. In certain practical scenarios where data on both the elastic modulus and normalized perfused area are unavailable, the elastic modulus is preferred due to its non-invasive monitoring capability using ultrasound shear wave elastography, in contrast to the more invasive contrast-enhanced ultrasound. Furthermore, considering feature importance, the model using the elastic modulus has higher prognostic performance compared to a model using the normalized perfused area. It can also be seen that by adding more input data that characterizes the tumors, the accuracy increases. Fig. 3a and b show the confusion matrix of two classification models: having as inputs the elastic modulus and perfused area (Fig. 3a) and having as inputs the elastic modulus and information on the treatment procedure (Fig. 3b). These two models were selected based on the unique and critical aspects they represent in evaluating treatment effectiveness. Model 1, utilizing elastic modulus and perfused area, likely focuses on the physical and functional changes in tissue responding to treatment, indicative of treatment's impact on tissue stiffness and blood flow. Model 2, incorporating elastic modulus and treatment procedure information, offers insights into how different treatment approaches affect tissue characteristics, considering the direct effects of various treatments.

**Table 1:** Classification models: Accuracy based on the input features used to characterize the tumors and classify them into responsive, stable, and non-responsive.

Input Data	Accuracy
Elastic modulus	0.889 ± 0.013
Normalized perfused area	0.789 ± 0.015
Elastic modulus & normalized perfused area	0.945 ± 0.008
Elastic modulus & treatment procedure	0.934 ± 0.006
Elastic modulus, normalized perfused area & treatment procedure	0.983 ± 0.005
Elastic modulus, normalized perfused area, treatment procedure & tumor type	0.983 ± 0.005

### Treatment characteristics

It has been well established that the use of mechanotherapeutics results in improved therapeutic effects, reduced tumor volumes (Fig. 2) and a higher probability for the tumor to be responsive to the cytotoxic therapy. However, it is of critical importance to compare the three different treatment groups that include mechanotherapeutics. Fig. 3c shows the relative tumor volumes of data belonging to these groups and their means were compared using ANOVA test. From this statistical test is shown that there are significant differences between the three groups. There is no significant difference between mechanotherapeutics & immunotherapy compared to mechanotherapeutics & chemotherapy & immunotherapy. However, both treatment groups are significantly different from treatment with mechanotherapeutics & chemotherapy. Therefore, we can conclude based on this data that mechanotherapy works better when combined with immunotherapy or chemo-immunotherapy.

### Mechanotherapeutics administration

**Fig. 4:**
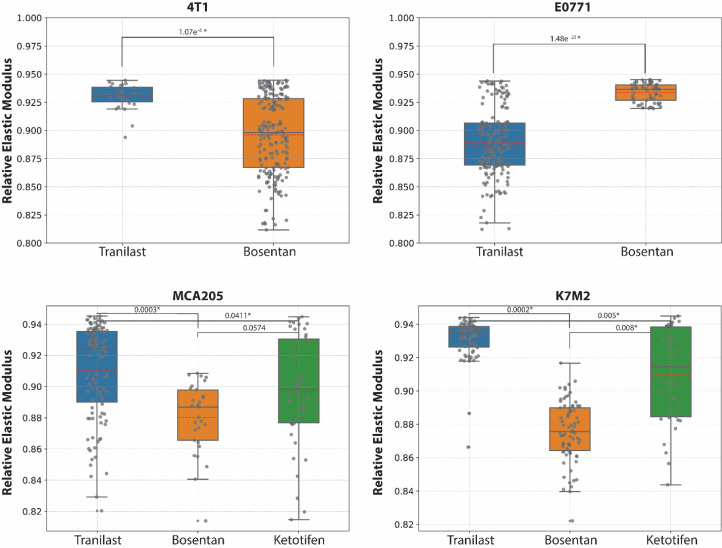
Mechanotherapeutics comparisons. Student's *t*-test p-values for 4T1 and E0771 are 1.0710^−6^ and 1.4810^−23^ respectively, indicating significant difference between the groups which is however reversed for the two cell lines. ANOVA p-values equal to 1.1310^−5^ and 5.3210^−38^ for MCA205 and K7M2 respectively.

Beyond comparing the various treatment groups, it is crucial to evaluate the different mechanotherapeutics themselves. This analysis was carried out by calculating the relative elastic modulus between measurements taken prior to the administration of mechanotherapeutics and the day of the initiation of the cytotoxic therapy (Fig. 1a). Fig. 4 illustrates that for all tumor types, the daily administration of mechanotherapeutics results in less stiff tumors and thus, reduced elastic modulus. However, when comparing different mechanotherapeutics, we observe significant differences between the groups but these differences are not consistent across all tumor types. For example, in the case of the 4T1 tumors, bosentan (1 mg/kg) appeared to be more efficient than tranilast (200 mg/kg), whereas the opposite was true for the E0771 tumors. To draw definite conclusions regarding which mechanotherapeutic works better for each tumor type, more experiments need to be conducted.

### Impact of mechanotherapeutic drugs across tumor types

In our study, we evaluated the effects of three mechanotherapeutic drugs—tranilast [20], [21], [22], bosentan [4,5] and ketotifen [19,23]—on different tumor models, including breast carcinoma, melanoma, and glioblastoma. Each drug demonstrated a distinct impact on tumor stiffness and perfusion, which in turn influenced the efficacy of subsequent chemotherapy and immunotherapy treatments. Tranilast was particularly effective in reducing tumor stiffness in the breast carcinoma models, leading to improved treatment outcomes. Bosentan showed a notable impact on improving tumor perfusion in the melanoma model, which was associated with a reduction in hypoxic regions and increased efficacy of immunotherapy. Ketotifen exhibited benefits across multiple tumor types by moderately reducing stiffness and improving perfusion, thereby facilitating a more uniform distribution of both chemotherapeutic and immunotherapeutic agents.

### Tumor growth prediction models

**Fig. 5:**
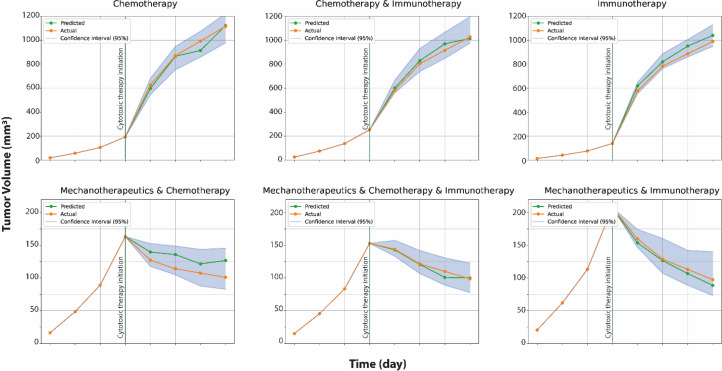
Regression analysis: Predicted tumor growth examples of tumors at the time of the cytotoxic therapy based on elastic modulus, normalized perfused area, treatment group and the tumor volumes at days 4, 7, 10 and the day that the cytotoxic therapy started. The analysis resulted in a Root Mean Squared Error of 97.79.

Fig. 5 shows the comparison between the actual tumor growth data and the predictions from the regression analysis for the six treatment groups. Specifically, the prediction of the projected tumor growth made on the day of the initiation of the cytotoxic therapy is presented with its confidence interval of 95 %. As expected, the volume predictions for longer times have a wider confidence interval. The observations in the test set to the predicted tumor growth was close to the actual one, the root mean squared error was 97.79 taking into account all treatment groups and individual tumors.

## Discussion

Our research explores the potential for personalized cancer treatment prediction considering the tumor stiffness and perfusion prior to the start of the treatment. It emphasizes the heterogeneity of tumors, highlighting that even within the same type of cancer, tumors can differ significantly. This variability poses a challenge for standard cancer therapies, which may be effective for some patients but not for others. To address this, the study focuses on identifying key tumor characteristics that could predict how a tumor responds to treatment. We compiled a comprehensive dataset of 1365 murine tumors from various cancer cell lines, including breast cancer, fibrosarcoma, osteosarcoma, and melanoma, and analyzed features, such as the elastic modulus and normalized perfused area.

Through statistical analysis and the application of machine learning models, the study determines the most important predictive features for treatment prediction. The importance of each characteristic was assessed, revealing that the elastic modulus and normalized perfused area were particularly significant. The study found that pre-treatment with mechanotherapeutics, which are aimed to alter the physical properties of tumors, could lead to better outcomes by reducing tumor volume and enhancing the effectiveness of subsequent cytotoxic therapies.

The study further highlights the promise of leveraging detailed tumor characteristics to predict treatment responses and to craft personalized therapeutic strategies in the realm of cancer care. The integration of mechanotherapeutics, along with the predictive power of tumor stiffness and perfusion, marks a significant stride forward in oncological practice. The outcomes of the study were encouraging, demonstrating that tumors could be effectively grouped based on their stiffness and perfused state. This categorization held true across all cancer cell lines tested, underscoring the reliability of these two characteristics as robust indicators for predicting responses to cytotoxic therapy.

We also developed various classification models to categorize tumors based on their response to therapy—responsive, stable, or non-responsive — using different sets of input features that characterize the tumors. This approach addresses the limitations of conventional treatment protocols, where certain invasive patient-related information, such as normalized perfused area, may not be readily available. Instead, our models leverage alternative features to make accurate prognostic outcomes. A XGBoost regression model was created to predict tumor volume progression, considering factors, such as the tumor's mechanical characteristics before cytotoxic therapy and the treatment protocol. Having insights into how a tumor will respond to therapy or how it's volume will progress provides the advantage of tailoring treatments to individual patients. For instance, it allows for adjustments in the mechanotherapeutics stage or faster initiation of cytotoxic therapy.

Discussing potential limitations and challenges in obtaining and analyzing tumor characteristics, particularly the elastic modulus and perfusion, is crucial for understanding the context and applicability of our findings. The measurement of the elastic modulus through SWE and perfusion via CEUS presents technical and biological challenges. These include variability in measurement precision as these methods depend on the operator, differences among ultrasound systems, and the inherent heterogeneity of tumors which may affect the consistency of these measurements. Additionally, the interpretation of these parameters can be complicated by the dynamic nature of tumor pathophysiology, where factors such as tumor size, location, and the surrounding tissue can influence the observed values. These challenges underscore the need for standardized protocols and cross-validation among studies to ensure the reliability and comparability of results.

The choice between supervised and unsupervised learning, logical (qualitative) or numerical (quantitative) approaches, is pivotal in machine learning [40,41]. Supervised learning, which XGBoost falls under, relies on labeled datasets to train algorithms that can predict outcomes or categorize data. This is in contrast to unsupervised learning, where algorithms identify patterns or groupings in data without pre-existing labels. Logical methods often categorize data into distinct classes based on rules, whereas numerical methods predict or quantify outcomes. Our selection of XGBoost and supervised learning was driven by the nature of our data and the specific objectives of our study, aiming to model and predict the efficacy of cancer treatments based on physiological indicators.

A significant limitation of our study is the exclusive focus on murine tumor models. While these models provide valuable insights into tumor biology and treatment responses, the direct extrapolation of our findings to human tumors presents considerable challenges. Murine models, despite their utility, do not fully capture the heterogeneity and complexity inherent in human cancers. Human tumors exhibit a wider range of genetic, cellular, and microenvironmental variations, which can significantly impact treatment outcomes. These differences can influence the efficacy of treatments that target mechanical properties of tumors, such as stiffness and perfusion. Therefore, while our results contribute to the understanding of tumor biomechanics and potential therapeutic strategies, caution must be exercised when applying these findings to clinical settings. Further research, including clinical trials, is necessary to validate the predictive value of tumor stiffness and perfusion in human cancer treatment and to adapt these biomarkers for clinical use. The classification models applied to breast cancer (4T1 and E0771), fibrosarcoma (MCA205), osteosarcoma (K7M2), and melanoma (B16F10) show promise for predicting treatment responses but require further validation across a broader spectrum of cancer types to ensure their widespread applicability in personalized cancer therapy.

## Conclusion

The results were promising, showing that tumors could be effectively classified into three groups based on their physical properties, and these classifications held true across all cell lines tested, but not so true for different drugs. This suggests that such characteristics are robust biomarkers for predicting responses to therapy. The study underscores the potential of detailed tumor analysis in improving the personalization and effectiveness of cancer treatments, advocating for the integration of these mechanical/imaging biomarkers into treatment planning. Such biomarkers could complement conventional biomarkers derived from genomic analysis to improve personalized treatment protocols.

## CRediT authorship contribution statement

**Demetris Englezos:** Writing – review & editing, Writing – original draft, Visualization, Validation, Software, Resources, Methodology, Investigation, Formal analysis, Data curation. **Chrysovalantis Voutouri:** Writing – review & editing, Writing – original draft, Visualization, Validation, Supervision, Software, Resources, Methodology, Investigation, Formal analysis, Data curation. **Triantafyllos Stylianopoulos:** Writing – review & editing, Writing – original draft, Supervision, Resources, Project administration, Methodology, Investigation, Funding acquisition, Conceptualization.

## Declaration of competing interest

The authors declare that they have no known competing financial interests or personal relationships that could have appeared to influence the work reported in this paper.

## Appendix Supplementary materials

Image, application 1
