# Supplementary material for: Machine learning analysis reveals tumor stiffness and hypoperfusion as biomarkers predictive of cancer treatment efficacy
Source: Transl Oncol. 2024 Mar 28;44:101944. doi: 10.1016/j.tranon.2024.101944 (PMC10990740; doi:10.1016/j.tranon.2024.101944)
Supplement: Supplementary file 1 [file mmc1.docx]

# Supplementary figures and Tables


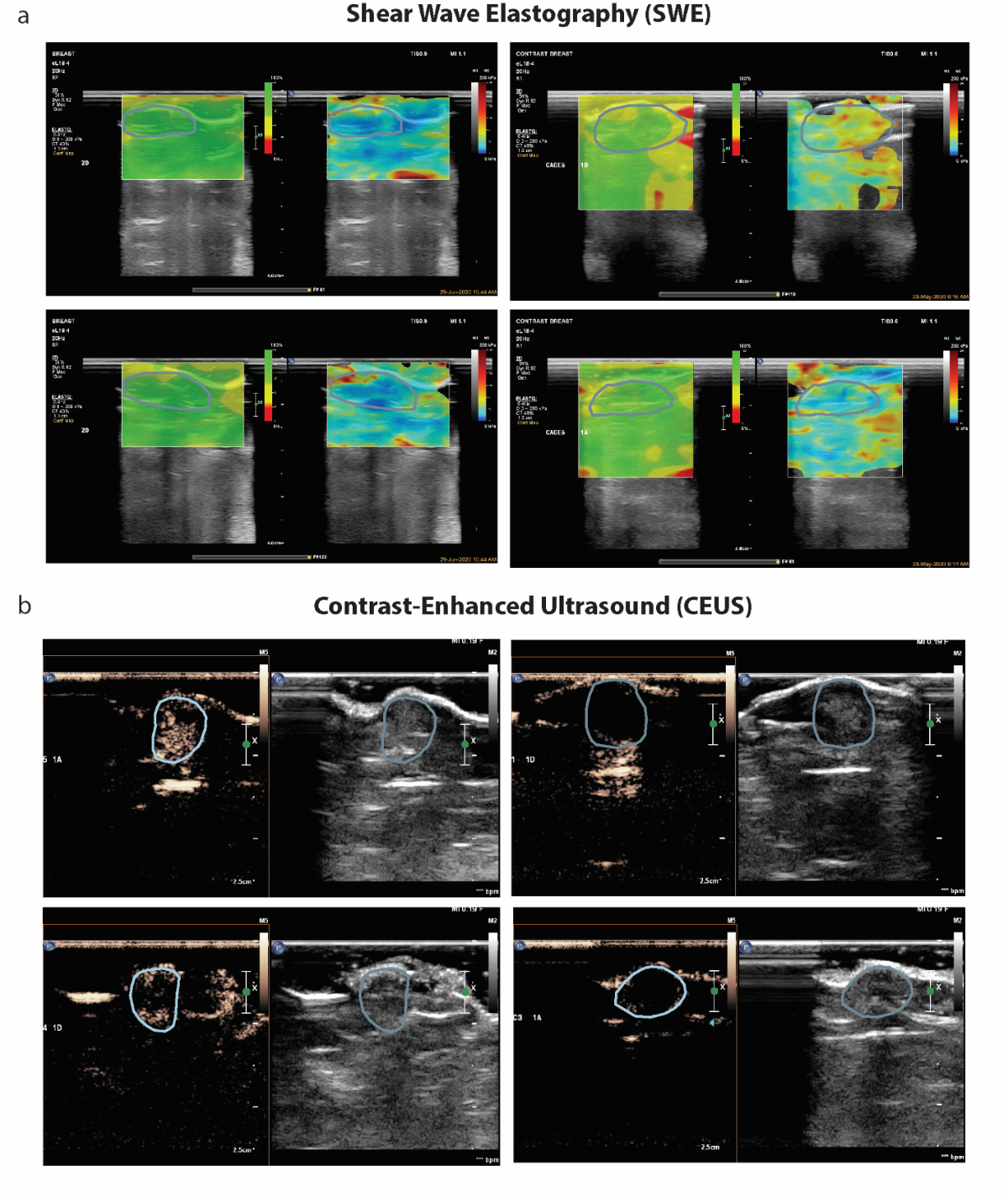


**Supplementary Figure 1**: The figure consists of four panels representing SWE images captured at four representative mice by treatment initiation: tumors reached a volume of 250-300mm3. Each panel displays a pair of images: the left image shows the confidence map of the SWE imaging, while the right image is the corresponding elastogram, indicating tumor stiffness in kPa. The color spectrum in the elastogram ranges from blue (indicating softer areas) to red (indicating stiffer areas), allowing for a visual assessment of changes in tumor stiffness over time. This additional description has been incorporated into the figure caption to aid readers in interpreting the images and understanding their relevance to our findings on the effects of treatment on tumor stiffness and response. CEUS Images: Similar to part a, these panels illustrate CEUS imaging at the same time. Each CEUS panel also includes a pair of images: the left image showcases the distribution of contrast agents within the tumor, indicating areas of blood flow, while the right image provides a standard ultrasound view for anatomical reference (B-mode). The solid lines again indicate tumor boundaries, facilitating an understanding of perfusion changes in relation to tumor growth and response to treatment.


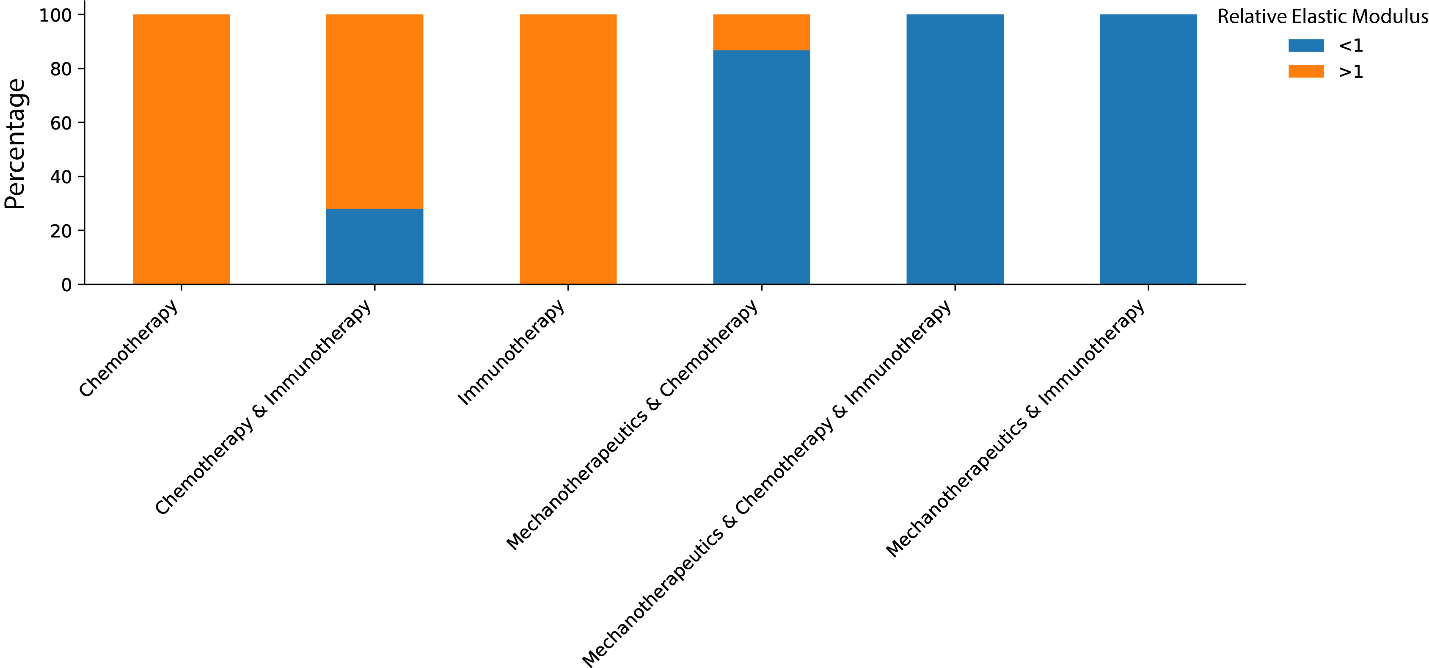


**Supplementary Figure 2**: The percentage of tumors with increased or decreased elastic modulus for the different treatment groups.


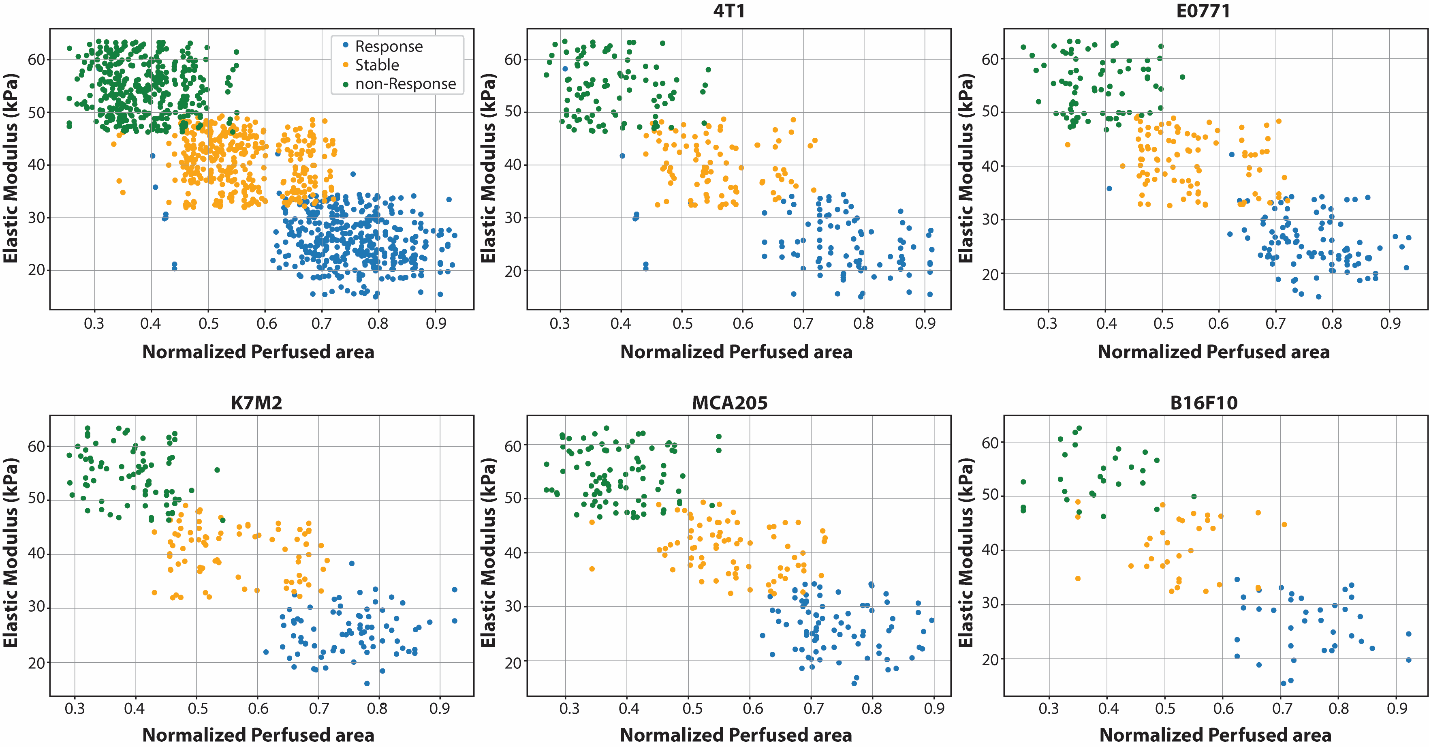


**Supplementary Figure 3:** **Tumor classification based on elastic modulus and normalized perfused area:** Tumors are classified into three classes: response (blue), stable (orange) and non-response (green), based on their elastic modulus and normalized perfused area characteristics. The first chart is a combination of the other five. The classification demonstrates that these two input features explain most of the data variability. The categorization of tumors remains consistent across all five tumor types (4T1, E0771, MCA205, K7M2 and B16F10) examined, validating the assertion that this separation holds true even when data from all tumor types are analyzed collectively.


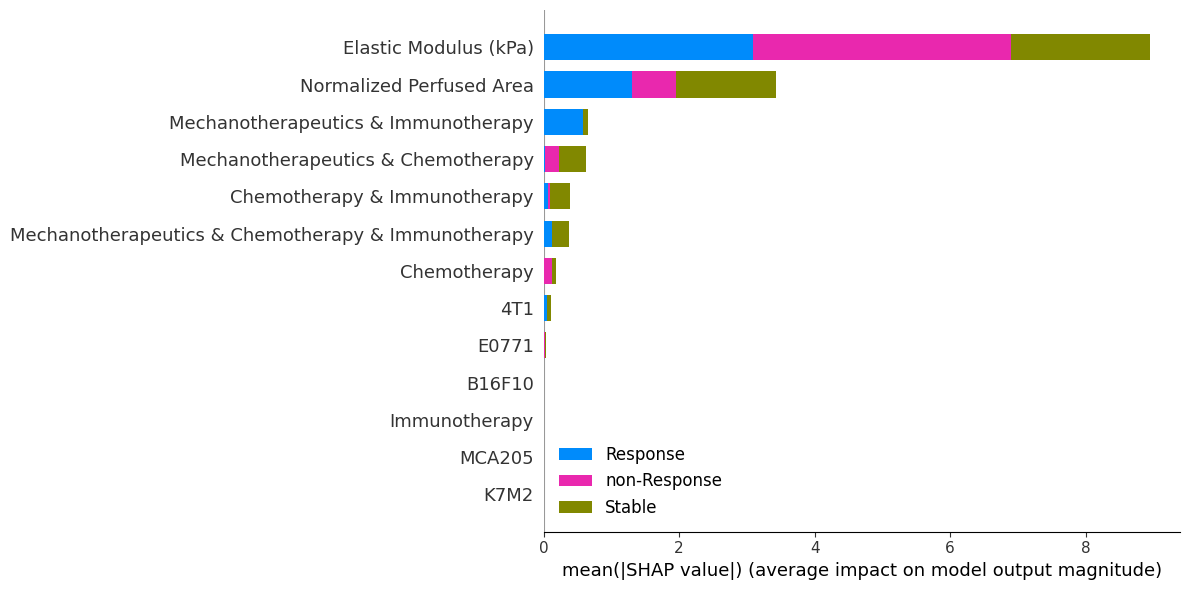


**Supplementary Figure 4:** **SHAP evaluation**. Interpretation and examination of predicted outcomes of the classification model using all features, shows the features that contributed the most. From up down they are graded from the most important statistically to the less significant.

**Supplementary Table 1:** **Feature Importance**. Feature coefficients and p-values for moving from response to stable.

| **Feature (“Response” to “Stable”)** | **Coefficient** | **P>\|z\|** |
| --- | --- | --- |
| Elastic modulus | 0.40 | >0.00 |
| Perfused area | -25.36 | >0.00 |
| Chemotherapy | -22.85 | 1.00 |
| Chemotherapy & Immunotherapy | 6.26 | 0.99 |
| Immunotherapy | -13.00 | 0.99 |
| Mechanotherapeutics & Chemotherapy & Immunotherapy | 4.53 | >0.00 |
| Mechanotherapeutics & Immunotherapy | 0.19 | 0.79 |

**Supplementary Table 2: Feature Importance**. Feature coefficients and p-values for moving from response to non-response.

| **Feature (“Response” to “non-Response”)** | **Coefficient** | **P>\|z\|** |
| --- | --- | --- |
| Elastic modulus | 0.71 | >0.00 |
| Perfused area | -56.97 | >0.00 |
| Chemotherapy | 10.51 | 1 |
| Chemotherapy & Immunotherapy | 7.79 | 0.99 |
| Immunotherapy | 13.75 | 0.99 |
| Mechanotherapeutics & Chemotherapy & Immunotherapy | -3.73 | 0.98 |
| Mechanotherapeutics & Immunotherapy | -29.43 | 0.99 |
